# Extreme Reproduction and Survival of a True Cliffhanger: The Endangered Plant *Borderea chouardii* (Dioscoreaceae)

**DOI:** 10.1371/journal.pone.0044657

**Published:** 2012-09-12

**Authors:** María B. García, Xavier Espadaler, Jens M. Olesen

**Affiliations:** 1 Pyrenean Institute of Ecology, Spanish National Research Council, Zaragoza, Spain; 2 Centre for Ecological Research and Forestry Applications, Universitat Autònoma de Barcelona, Bellaterra, Spain; 3 Institute of Bioscience, Aarhus University, Aarhus, Denmark; Norwegian University of Science and Technology, Norway

## Abstract

Cliff sides are extreme habitats, often sheltering a rich and unique flora. One example is the dioecious herb *Borderea chouardii* (Dioscoreaceae), which is a Tertiary, tropical relict, occurring only on two adjacent vertical cliffs in the world. We studied its reproductive biology, which in some aspects is extreme, especially the unusual double mutualistic role of ants as both pollinators and dispersers. We made a 2-year pollination census and four years of seed-dispersal experiments, recording flower visitors and dispersal rates. Fruit and seed set, self-sowing of seeds, seedling recruitment, and fate of seedlings from seeds sowed by different agents were scored over a period of 17 years. The ants *Lasius grandis* and *L. cinereus* were the main pollinators, whereas another ant *Pheidole pallidula* dispersed seeds. Thus ants functioned as double mutualists. Two thirds of all new seedlings came from self-sown seeds, and 1/3 was dispersed by ants, which gathered the seeds with their oil-rich elaiosome. Gravity played a minor role to dispersal. Both ant dispersal and self-sowing resulted in the same survival rate of seedlings. A double mutualism is a risky reproductive strategy, but *B. chouardii* buffers that by an unusual long–term demographic stability (some individuals exceed 300 years in lifespan) and its presence in a climatically very stable habitat, inaccessible to large herbivores. Such a combination of traits and habitat properties may explain the persistence of this relict species.

## Introduction

Ants are ubiquitous in nature, playing key ecological roles, not only in tropical and temperate ecosystems [1], but also in harsh environments like deserts and alpine habitats [2]. Two of their ecosystem functions are pollination and seed dispersal.

Ants are frequent floral visitors [2], but are often regarded as inefficient pollinators because of their small body size, short foraging range, and secretions from their metapleural glands, which may reduce pollen viability (“the antibiotic hypothesis”) [3], . However, several reports demonstrate the importance of ants as pollinators, e.g. in a population of an alpine plant, Gómez and Zamora [5] showed that one of the flower–visiting ant species enhanced plant female fitness more than all the 39 winged insect visitor species together. The high frequency of ant visits and their presence during the entire flowering period may be reasons for the pollinatory success of this single ant species [6], [7]. Generally, ant pollination may be most common where abiotic conditions for flying insects are adverse, e.g. in mountains and deserts [2], .

Despite that ants are well known as seed predators or harvesters [9], they also play an important role as seed dispersers. Seed dispersal by ants is known from at least 3,000 plant species but may be found in four times as many [10]. It has evolved independently in more than one hundred lineages, which subsequently diversified more than their non–ant–dispersed sister lineages [10]. This accelerated diversification rate was kicked off by a key innovation, the elaiosome, i.e. a small food body attached to seeds, which attracts ants [10]. The elaiosome is lipid–rich and nitrogen–poor. Typically, ants harvest the seed with its elaiosome and carry it back to the nest, where they bite off the elaiosome and feed it to their larvae. Afterwards, the “garbage”, that is the seed without elaiosome, is deposited either inside the nest or outside in a refuse pile [10]. This behaviour may enhance plant fitness by moving seeds to seed predator–free and nutrient–rich sites suitable for germination or to a seed bank during periods of abiotic stress, reducing intraspecific competition [11], [12]. Ants are, however, probably mediating the shortest seed flow of any animal disperser, viz. only 0.01–77 m [13].

For most plants, the pollinator and seed–disperser fauna differ from each other [14]. However, in habitats poor in animal diversity, such as deserts, islands, and mountains, plants may use the few resources available, and consequently, evolve towards double mutualism, i.e. to use the same animals as both pollinators and seed dispersers. A few examples are known, e.g. several island plants have lizards, birds or flying foxes as their double mutualists [15]–.

In mountains, cliff sides constitute “ecological islands”, and they are among the resource–poorest habitats in the world [18]. In recent years, they have received increasing attention by ecologists, [18], [19]. Besides their steep orientation, cliff sides have ecological characteristics that distinguish them from other habitats: low availability of nutrients, very limited space for root development and scarce possibilities for biotic recruitment. Species able to live under such conditions, however, may be protected against climatic extremes (for example in deep canyons), large herbivores and most human effects. The fact that rock plants often are small and long–lived, but make up stable populations, suggests their rate of recruitment and mortality is very low [20], [21]. However, several aspects of their life history are enigmatic. For example, how do rock plants get their seeds dispersed to safe crevices, avoiding that their populations after a few generations “slide” down the cliff side and go extinct?

Here, we address this question by studying the role of ants to the pollination and seed dispersal of one of the most ancient and endangered European plants, *Borderea chouardii* (Gaussen) Heslot (Dioscoreaceae) [22], [23]. It is a small, strictly cliff–growing or rupicolous plant, occurring on shady, vertical limestone cliffs and overhangs in the central Spanish Pyrenees. The species belongs to a small dwindling element of relicts from a long gone Tertiary tropical flora, and it has the highest conservation priority in Europe (European Commission, Environment: Habitats Directive; Council Directive 92/43/EEC of 21 May 1992 on the conservation of natural habitats and of wild fauna and flora). However, the life span of its individuals is very unusual, being one of the longest ever recorded for any non–clonal herb, viz. >300 years [21]. Given the difficulties to conduct standard studies in the habitat of the plant, we accumulated detailed observations and carried out experiments in both the field and the lab for up to 17 years, to demonstrate a double mutualistic interaction between the plant and its pollinating and seed–dispersing ants. Finally, we discuss the role of these ants in relation to the *B. chouardii*’s astonishing long–term persistence.

## Materials and Methods

### The Plant

**Figure 1:**
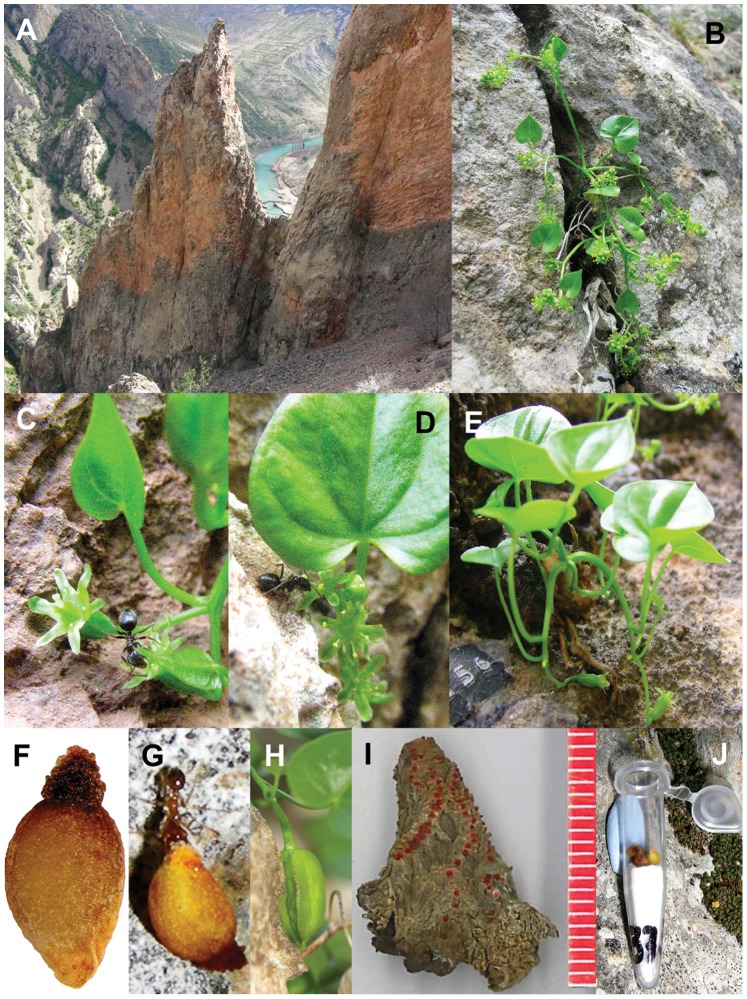
*Borderea chouardii*. (A) Topography of the habitat (Sopeira, Spanish Pyrenees), (B) flowering male plant, (C) flowering female plant with a visiting ant (*Lasius*), (D) male flowers with a visiting ant (*Lasius*), (E) female plant with two fruits, one at a crevice in the cliff wall (self sowing), (F) seed with elaiosome coating, (G) vial containing seeds *in situ*–for cafeteria experiments (Experiment I, see Materials and Methods), (H) ant removing a seed (Experiment I), and (I) tuber with old leaf scars marked with a red coloration, each dot corresponds to one year, and a ruler shows its size in mm.

Worldwide, *Borderea chouardii* is known from one single population (Fig. 1A), located 850 m a.s.l. in the Spanish Pyrenees [24]. It was discovered only 60 years ago, probably due to its occurrence in a topographically complex and inaccessible area. Here its entire habitat covers a few thousand m^2^. The plant grows on two vertical cliff walls and even upside down under the “ceiling” of a short cave without receiving any direct sunlight. The walls are stable, and only a few downfalls of stone chips have been recorded during the study period 1995–2011. A demographic monitoring project of a large sample of plants was initiated in 1995, using scaffolding and climbing gear [21].

The species is a small, dioecious geophyte of <2 g in individual biomass, with twice as many male as female plants. The tuber grows in small crevices in the wall without any vegetative propagation. Male plants produce more and smaller leaves, and flower at an earlier age and more profusely than females (Fig. 1B,E; M. B. García *unpublished data*). Both sexes have small, green flowers with tiny amounts of nectar. The ovary has six ovules. Floral pedicels are often close to the rocky wall (Fig. 1C, D) and once fertilized, female flowers turn towards the wall. The pedicel of ripening fruits may even elongate pressing the fruits into a crevice, where the seeds become released (Fig. 1E). This is termed self-sowing [21]. Seeds are brown, ovoid shaped, about 3 mm long, and have a tough oily coat, which becomes very dense at the apex. This coating and the dense apex function as an elaiosome (Fig. 1F).

### Pollination and Reproductive Success

In order to identify flower visitors of *B. chouardii*, we spent 76 hours observing plants for flower–visiting insects, viz. 61 hours and 15 hours in 2008 and 2009, resp., or 53 (69%) and 23 hours (31%) observing males and females, resp. These focal plants were chosen randomly within the narrow vertical zone on the cliff wall of the population. Flower–visitation observations were made from 17–30 May, covering the entire flowering period. We did 397 censuses, each lasting 10–15 min at both groups and solitary plants. Gender, and numbers of open flowers per plant and flower visits by insects were recorded. Whenever in doubt about taxonomic status of a visitor it was sampled for later identification. *t*-tests were used to compare the frequency of visits to male and female plants and flowers. The likelihood of wind pollination was assessed by placing microscopic slides with glycerol on the walls 20 cm from a flowering male, and later inspecting slides for pollen.

**Figure 2:**
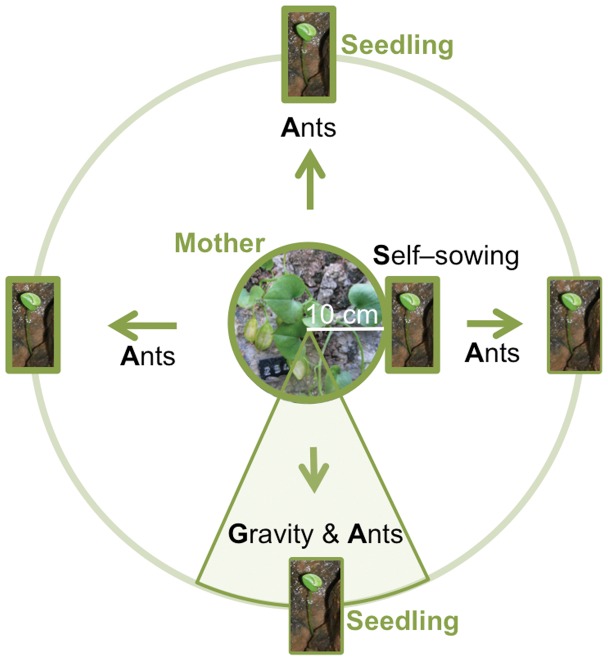
Seed–dispersal modes of *Borderea chouardii*. Dispersal by gravity (G) is assumed to take place within a circular section of 45° below a mother and >10 cm away from the mother. Dispersal by ants (A) takes place in all directions and >10 cm away from the mother, and self–sowing (S) is restricted to a circular area of a radius of 10 cm and the mother plant as its center.

Annually from 1995 to 2011, fruit set (the ratio of numbers of fruits : flowers), and seed set (the ratio of numbers of seeds : 6 ovules in ripening fruits) were estimated [21]. Fruit ripening happened in September and seeds were either dispersed by ants (*A*), gravity (*G*) or through “self-sowing” (*S*) (Fig. 2).

### Seed Dispersal

Self-sowing was estimated between 1995–2011 as the percentage of ovaries growing within crevices. The rest of the fruits ripe mostly in contact with the rocky surface, where the dry capsules open and may contain up to 6 seeds. During the fruit–ripening period of *B. chouardii*, three ant species were observed at the study site: *Pheidole pallidula*, *Lasius grandis* and *L. cinereus*. In order to determine the role and importance of ants as seed dispersers, and given the difficulty of monitoring *in situ* seed dispersal and seedling recruitment, we gathered information from a set of experiments.

#### Experiment I

We made an *in situ*–“cafeteria” experiment to test the hypothesis that the ants we observed in the population were seed dispersers, and that the elaiosome was the unit of attraction (Fig. 1F). The experiment was commenced at the onset of the natural seed release in the population. Forty vials (1 cm wide, 4 cm deep) were glued to the cliff wall (Fig. 1G) and in each vial, we placed either (a) six seeds with elaiosome (2008 and 2011), (b) three seeds with elaiosome + three elaiosomes + three seeds without elaiosome (2009; for protocol details see [25]), or (c) one open fruit containing six non-shed seeds (2010). Every 5–10th day during six weeks, vials were inspected and numbers and kinds of removed items were scored. In 2008 and 2009, a few vials were lost or got filled with rainwater, leaving 37 and 35 vials for analysis, respectively. Generalized linear models (*glm* function, R Core Development Team 2011) were used to test for the preference of items by ants.

#### Experiment II

We also wanted to know if seeds of *B. chouardii* were particularly attractive to ants compared to other species with or without elaiosome. Given the difficulty to find nests in the population, in September 2008 we used 12 natural nests *P. pallidula* (the only species observed to remove seeds from *in situ–*vials; Fig. 1H) for a food–choice experiment in another location. Seeds of four plant species were offered to the ant: (1) *B. chouardii*, (2) its congeneric, the scree plant *B. pyrenaica* (its seeds have an oily coating too but no distinct elaiosome at their apex, (3) the rupicolous *Sarcocapnos enneaphylla* (seeds with elaiosome and co–occurring with *B. chouardii*), and (4) the partially rupicolous *Silene acaulis* (seeds without elaiosome and not co–occurring with *B. chouardii*). A group of four seeds (one of each species) was placed 10 cm from the entrance of each nests. During 10–min intervals of observation, the behaviour of *P. pallidula* workers to the presence of seeds was recorded. When leaving their nest, ants always had the possibility to choose among all four species of seed, because if an ant removed a seed, it was immediately replaced. Possible ant responses were: “not removed” (either not interested in any seed, or seed examined but not removed), and “seed removed”. Total number of ant responses was 514. Generalized linear mixed models (GLMM) were used to model the preference of ants to specific seeds. “Nest” was treated as a random factor, and the *lme4* function in R was used [26].

#### Experiment III

Finally, we tested how the two ant genera (*Pheidole* and *Lasius*) treated seeds of *B. chouardii*. Since it is not possible to observe the handling of seeds within nests on the wall, we studied this experimentally in the lab. A total of 30 seeds of *B. chouardii* were placed in front of the entrance to five artificial nests of *Pheidole pallidula*. We recorded if seeds were introduced to the nest, and one week later if they still had the elaiosome, were without elaiosome or were destroyed by predation.

In November 2010 after the fruiting season of *B. chouardii*, we carried out one further *ex situ–*experiment with the two *Lasius* species. Once a week, three artificial nests of *L. grandis* (young colonies: 1 queen +10–12 small workers) and *L. cinereus* (>100 workers +50–100 larvae) were offered six seeds of *B. chouardii*, and additionally, fed an artificial diet [27]. In mid–December, we had to let the nest hibernate, and the six nests were placed outdoor. In mid–February, they were returned to the laboratory (18–22°C). Position of seeds (out/inside the nest) and condition (elaiosome present/absent) were scored immediately before hibernation, and four weeks after hibernation period. Hibernating larvae began to develop normally after hibernation and to pupate.

The Environmental Service of the Regional Governement of Aragón gave the permit to do *in situ* and *ex situ* experiments involving seeds. The regional government is the responsible authority for the recovery plan implemented in 1995.

### Successful Seed Dispersal and Survival of Seedlings

During 1995–2011, we studied the relative importance of different seed dispersal modes by recording the position of all new seedlings (1-year old) in the monitored area, and estimated survival probability. These represent successful dispersal events. We hypothesized that dispersal could take place in three ways: by self-sowing (*S*), ant (*A*), and gravity/rain (*G*). *S* included seeds dispersed <10 cm, i.e. within the circumference of the pedicels of a female plant (same crevice or a close one reachable by fruiting pedicels; see Fig. 1E). *A* included seeds dispersed >10 cm from nearest female plant but not directly below a female. Finally, *G* included seeds dispersed >10 cm and directly below the nearest female (Fig. 2). In order to distinguish between *A* and *G* in the field, we took into account the direction from each seedling to nearest female. If a female was growing directly above the seedling (within a circular section of ±23°) and being >10 cm away, the dispersal was scored as *G*, if not as *A* (Fig. 2). Nevertheless, ants can also move seeds downwards, and thus a small fraction on *G-*seedlings could actually come from *A-*. Dispersal rates were adjusted accordingly (see the results section).

The survival probability of all seedlings recorded over 17 years of monitoring was compared among different dispersal modes (*S, A, G*) by generalized linear models (*glm* function in R, binomial distribution).

## Results

### Pollination and Reproductive Success

Habitat and habit of *B. chouardii* are shown in Fig. 1. Population sex ratio, i.e. the numbers of individual male to female plants, was 2.2 (*N* = 346; Table 1). Male and female plants had 44.4±47.1 (mean ± SD; *N* = 239 plants, range 1–244) and 4.4±3.7 (*N* = 107 plants, range 1–23) simultaneously open flowers, respectively. Thus open male flowers were (2.2×44.4/4.4 = ) 22 times as frequent as female flowers in the population. Male plants received the same number of visitors but more visits (plant^−1^ hour^−1^) than females (*t–*test (ln(*x* +1)–transformed data): *t* = 1.41, *P* = 0.16 (visitors); *t* = 2.51, *P* = 0.01 (visits); *N* = 397 plant visitor/visit census), whereas individual male and female flowers had the same visitation rate (visits flower^−1^ hr^−1^) (*t–*test (ln(*x* +1)–transformed data): *t* = 1.71, *P* = 0.09; *N* = 397 floral visit census).

**Table 1:** Flower visitation of *Borderea chouardii.*

	Male	Female	Male : female
Observation time (hrs)	53	23	2.3
No. observed plants	239	107	2.2
No. flowers	8329	456	18.3
No. flowers/plant	44.4	4.4	10.2
Obs. time (min)/plant	13.2	12.9	1.0
Total no. visitors	47	11	4.3
Total no. ants	33	9	3.7
No. visitors/plant/hr	1.0	0.8	1.4
No. visits/plant/hr	3.7	1.0	3.6
No. visits/flower/hr	0.1	0.3	0.3

During the entire flowering season (17–30 May) in 2008 and 2009, we observed a total of 58 flower visitors (Table 1). Seventy percent were ants: *Lasius grandis* (59% of all ant records), *L. cinereus* (11%), *Camponotus cruentatus* (11%) and unidentified Formicidae species (19%) (Fig. 1C,D). Besides ants, a Collembola species (seven visitors), a parasitic Hymenoptera species (five visitors), a Coleoptera species (two visitors), and a Neuroptera species (one visitor) were observed in the flowers. Ants constituted 82% of all visitors to female flowers because they received less visits from non–ants. In the wind–pollination experiment, no *B. chouardii* pollen at all were found on any microscopic slide (*N* = 10 slides).

**Figure 3:**
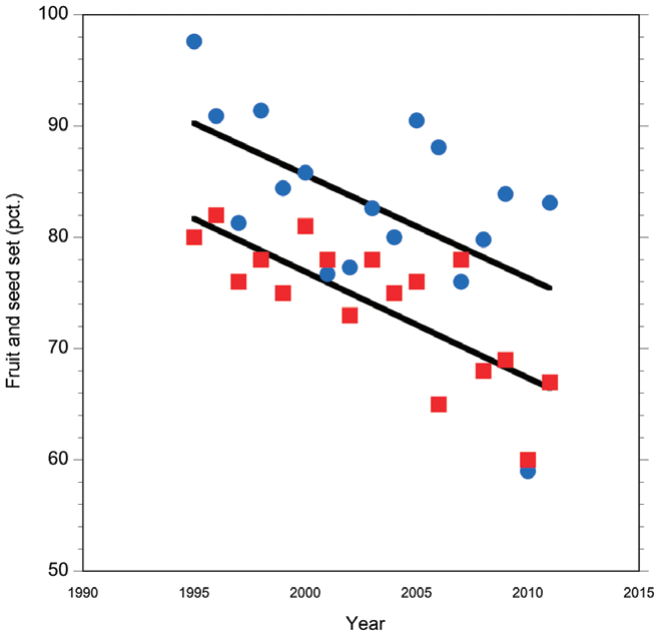
Reproductive success of *Borderea chouardii* over 17 years. Reproductive success was estimated as fruit set of individual plants (blue dots; percentage of fruits to flowers) and seed set (red squares; percentage of seeds to ovules).

Across 17 years, mean fruit set was 82.8% ±8.5% (average ± SD; *N* = 3,287 flowers, range 59%–98%; Fig. 3), but fruit set has been declining (*R* ^2^ = 0.30; *P* = 0.02). In fruits seed set was 74.1% ±6.2% (average ± SD; *N* = 2,761 fruits, range 60%–82%; Fig. 3), and it also declined significantly (*R* ^2^ = 0.61; *P* = 0.001).

### Seed Dispersal

Only 8.4% ±3.9% of the 2,568 fruits examined between 1995–2011 were self-sowed. Therefore, most seeds produced in the population were eventually released on the air unless harvested by ants or retained in crevices when rolling down by gravity.

#### Experiment I

In September during fruit ripening, only one ant species (*Pheidole pallidula*) was observed to remove seeds from vials and bringing them into nearby crevices. The other two ants recorded in the monitoring area (*Lasius cinereus, L. grandis*), the same that visited flowers four months earlier, were observed in the cliff side but no interaction with the plant or vials was recorded. Nevertheless, in spring 2011, two seedlings were observed to root in active nests of *Lasius* spp.

**Figure 4:**
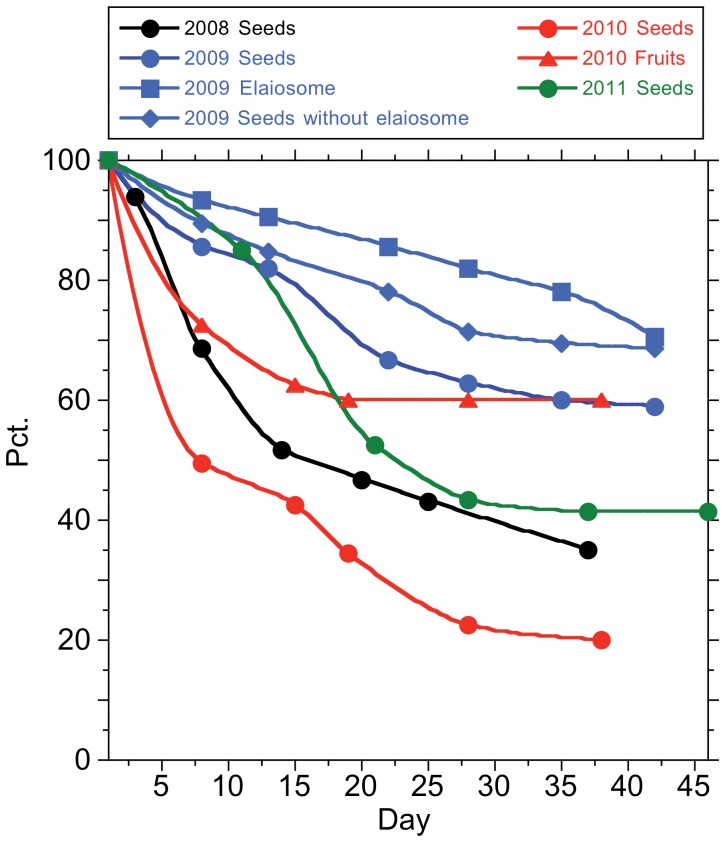
Removal of different kinds of seed items of *Borderea chouardii* from *in situ* cafeteria experiments. Categories are seeds with elaiosome (2008, 2009, 2011), seeds without elaiosome (2009), individual elaiosomes (2009) and entire open fruits containing 6 seeds (2010).

In the *in situ–*cafeteria experiment, both seeds and entire fruits were removed from vials (Fig. 1G, 4). Seed removal rate in cafeteria experiments varied between 40–80% of all seeds offered in vials during six weeks (Fig. 4). Seeds with elaiosome were removed more intensively in 2008 than in 2009, but not significantly faster than elaiosomes alone (*Z* = 1.73, *P = *0.08) or seeds without elaiosome (*Z = *1.43, *P = *0.15).

#### Experiment II

**Figure 5:**
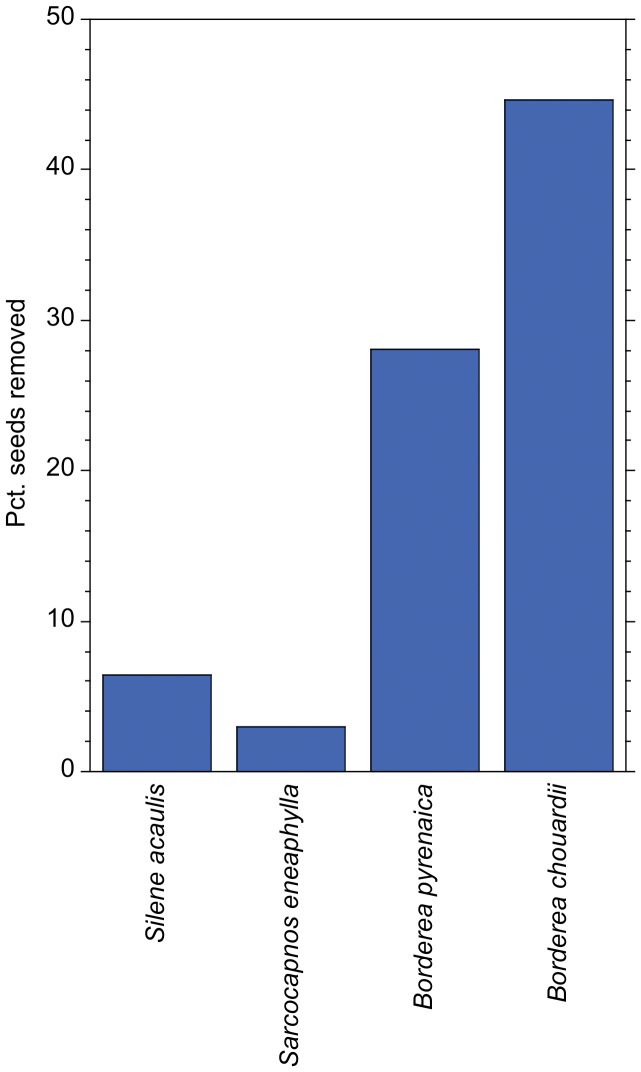
Frequency of seed removal of four plant species in natural nests of the ant *Pheidole pallidula*. Bch = *Borderea chouardii*, Bp = *Borderea pyrenaica*, Se = *Sarcocapnos enneaphylla*, and Sa = *Silene acaulis*.

The *ex situ*–cafeteria experiment with *Pheidole pallidula* clearly showed that it preferred seeds of *B. chouardii* to those of any of the other three species (*B. chouardii vs. B. pyrenaica*: *Z* = 3.60, *P* = 0.0003; *vs. Sarcocapnos enneaphylla*: *Z* = 5.77, *P = *0.0001; and *vs. Silene acaulis*: *Z* = 6.30, *P = *0.0001). However, seeds of both *Borderea* species were preferred to seeds of the other species (Fig. 5).

#### Experiment III

All 30 seeds of *B. chouardii* placed in front of *P. pallidula* laboratory nests were harvested. Sixty–three percent were predated, while the rest were discarded intact. Both *Lasius grandis* and *L. cinereus* left the elaiosome and the seed coat untouched, i.e. all seeds remained intact. All seeds offered to *L. cinereus* remained outside the nest before and after hibernation. The response of *L. grandis* in young nests before hibernation varied. Before and after hibernation, 1/3 of the nests had seeds inside the nests.

### Successful Seed Dispersal and Survival of Seedlings

During 1995–2011, the estimated proportions of *S*–, *A*– and *G*–seedlings were 51%, 39% and 10% respectively (*N* = 139 seedlings). Our *G-*seedlings, however, could contain some *A-*seedlings too because ants can move seeds downwards in the crevice. If dispersal away by ants from the mother plant was random, and we name *a* as total *A* and *g* as total *G*, we would expect for non self-sowed seedlings: (100%–51%) = 49% = *a* + *g*, and for seedlings 10 cm away below a female: 10% = *a* *(45°/360°) + *g* (Fig. 2). Thus our best estimate of the proportion of seedlings dispersed by *g* becomes 4.4% and that of *a* becomes 44.6%.

Survival probability of 1-year old seedlings was 63% after *A* (*N* = 43) and 70% after *S* (*N* = 69) and the difference was non–significant (*Z* = 0.74, *P = *0.46).

## Discussion

*Borderea chouardii* is dioecious, which precludes any self–pollination. In addition, we ruled out wind–pollination experimentally, leaving animal pollination as our only remaining option. However, in spite of many hours of observation of flowers, only three species of ants (*Lasius grandis*, *L. cinereus*, and *Camponotus cruentatus*) were observed as visitors attracted by the nectar, besides a few collembolas and parasitic hymenopterans. *Borderea chouardii* does have several characteristics associated with ant pollination, especially easily accessible nectar, low growth form, and small flowers being less attractive to larger insects [2], [28]. Thus we conclude that *B. chouardii* is ant–pollinated, but that the visitation rate of ants is as low as *c.* 1 ant/plant/hour. Its reproductive success is high, although we observed a steady decline over the years. The congeneric *Borderea pyrenaica*, also restricted to the Pyrenees, but growing on screes, is ant–pollinated as well and has a similar seed production [6]. Here, an experimental study demonstrated that pollen transported by its ant pollinator *Leptothorax tuberum* was viable [6]. Thus, ants are successful pollinators of the only two species in this tropical relict, montane, endemic plant genus.

Seeds of *B. chouardii* were dispersed by self-sowing, ants, and/or gravity. Self-sowing was a likely dispersal mode because of the unusual skototropic behavior, i.e. elongation towards nearby dark crevices, of fruiting pedicels [29]. Successful skototropic events, although not frequent (9%), ended up in fruits ripening within crevices, where they dehisced and the seeds were released [Fig. 1E].

*A priory*, ants were seed–disperser candidates because of the elaiosome of the seeds, which is unique in the family Dioscoreaceae (mostly wind-dispersed), and is, in general, a key adaptation to ant–seed dispersal. Ants were never directly observed gathering the seeds from plants on the cliff, but three species (*Lasius cinereus, L. grandis, Pheidole pallidula*) were observed on the wall near plants with ripe fruits. The latter species was observed to remove seeds from vials and bringing them into nearby crevices, and some seedlings were found to be rooted in active *Lasius* nests. Skototropism also increased ant’s probability to encounter fruits in the cliff, and resulted in an increased and less variable ambient temperature to the fruits due to the higher specific heat capacity of the rock compared to air. This could accelerate ripening, which might become increasingly important with the decline in ant abundance on the rock walls in the early autumn. This late-seasonally ant–seed dispersal of *B. chouardiii* seems to be unique among temperate ant–dispersed plants in general [30], which most often are fruiting in spring or early summer.

Seeds with or without elaiosome were removed from the vials with similar rate (Experiment I). *Pheidole pallidula* showed a preference for the seeds of *Borderea chouardii* compared to seeds with elaiosome of other species, but also harvested seeds of its congener *B. pyrenaica*, without elaiosome but with the same kind of oily coat (Experiment II). *P. pallidula* predated 2/3 of all *B. chouardii* seeds collected and left 1/3 intact (Experiment III). We conclude that *P. pallidula* was a seed disperser of *B. chouardii*, but its price in predated seeds for its mutualistic services was probably high [14], [31]. *Lasius* species also harvested the seeds of *B. chouardii*, did not predate them, and their interest in the elaiosome was uncertain (Experiment III). This ant genus is a well–known group of seed dispersers [30], [32], and it must disperse seeds of *B. chouardii* because some seedlings have been found to grow in its nests. In contrast to *Pheidole, Lasius* ants have never been recorded as seed predators (according to the FORMIS 2009 database) [33].

Finally, gravity was also a likely mode of dispersal because of the vertical habitat. However, it seems to be of minor importance given the low frequency of new recruited seedlings by this dispersal mode. The reason is probably the combination of skototropism and ants, together with the low chance of being retained in the few crevices available when seeds are released.

Our conclusion is that ants serve as both pollinators and seed dispersers of *B. chouardii*. This is one of the very few records of ants as double mutualists. However, the species runs a double jeopardy putting all its stakes on just one kind of mutualist. Only a very long-lived plant can reduce that risk, because longevity confers demographic stability and increases the independence from recruitment [28], [29]. In fact, *B. chouardii* probably holds the astonishing world record in individual lifespan among non-clonal plants: >300 years (Fig. 1I). About 1,000 plants may grow on the monitored area, i.e. about 700 males and 300 females. During 17 years of population monitoring, 139 seedlings were recorded. That is a mean of only 8.2 per year or 0.03 per female and year. In spite of this low recruitment, the demographic dynamics of the species is one of the most stable known among herbaceous plants [21].

Rocky habitats are widespread, but the ecology of their inhabitants is poorly known because of obvious accessibility problems. Consequently, they are among the least disturbed places on our planet, and play a major role as natural reserves for many rare and endemic species [18]. Rocky habitats, therefore, are of outstanding value to conservation of biodiversity. However, rock–living plants experience strong selection from especially nutrient deficiency, shortage of recruitment sites and the detrimental consequences of gravity to seed dispersal. Ants can mitigate this by offering mutualism services and nutrient-rich recruitment sites.
